# High Neutrophil-to-Lymphocyte Ratio Facilitates Cancer Growth—Currently Marketed Drugs Tadalafil, Isotretinoin, Colchicine, and Omega-3 to Reduce It: The TICO Regimen

**DOI:** 10.3390/cancers14194965

**Published:** 2022-10-10

**Authors:** Richard E. Kast

**Affiliations:** IIAIGC Study Center, Burlington, VT 05408, USA; richarderickast@gmail.com

**Keywords:** cancer, inflammation, myeloid derived suppressor cells, neutrophil-to-lymphocyte ratio, NLRP3 inflammasome

## Abstract

**Simple Summary:**

Several elements that are composed of, or related to, neutrophils, have been shown to inhibit strong immune responses to cancer and promote cancers’ growth. This paper presents the collected data showing these elements and how their coordinated actions as an ensemble facilitate growth in the common cancers. The paper goes on to present a drug regimen, TICO, designed to reduce the cancer growth enhancing effects of the neutrophil related elements. TICO uses four already marketed, readily available generic drugs, repurposed to inhibit neutrophil centered growth facilitation of cancer.

**Abstract:**

This paper presents remarkably uniform data showing that higher NLR is a robust prognostic indicator of shorter overall survival across the common metastatic cancers. Myeloid derived suppressor cells, the NLRP3 inflammasome, neutrophil extracellular traps, and absolute neutrophil count tend to all be directly related to the NLR. They, individually and as an ensemble, contribute to cancer growth and metastasis. The multidrug regimen presented in this paper, TICO, was designed to decrease the NLR with potential to also reduce the other neutrophil related elements favoring malignant growth. TICO is comprised of already marketed generic drugs: the phosphodiesterase 5 inhibitor tadalafil, used to treat inadequate erections; isotretinoin, the retinoid used for acne treatment; colchicine, a standard gout (podagra) treatment; and the common fish oil supplement omega-3 polyunsaturated fatty acids. These individually impose low side effect burdens. The drugs of TICO are old, cheap, well known, and available worldwide. They all have evidence of lowering the NLR or the growth contributing elements related to the NLR when clinically used in general medicine as reviewed in this paper.

## 1. Introduction

This paper aims to extend application of the movement repurposing already-marketed non-oncology drugs as adjuncts to current cancer treatments. The presented regimen, TICO, (tadalafil, isotretinoin, colchicine, omega-3 fatty acids) uses four FDA approved non-oncology drugs to partially reverse the characteristic immunosuppressive elements common in glioblastoma and in cancers generally.

First the paper will review the collected evidence that (A) the circulating, blood neutrophil-to-lymphocyte ratio (NLR) is elevated in all the common metastatic cancers, (B) that a higher NLR is associated with shorter survival across the common cancers, (C) that the NLR itself is retarding of immune responses, (D) that elevated NLR is associated with elevated numbers and function of myeloid-derived suppressor cells (MDSC), and that (E) normal neutrophils commonly also contribute to malignant growth in most cancers.

Secondly, this paper presents data on four already marketed generic drugs from general medical practice showing that they individually can lower the NLR in humans.

Neutrophils are viewed today as a “double edged sword” in that we have data on an anti-malignant cell activity of neutrophils, as well as a (much larger) database on a growth facilitating role of neutrophils contributing to malignant growth [1,2,3]. The latter is the subject of this paper.

Three nuances of note here: (A) A given cell, signaling system, or physiological element need not be either cancer growth facilitating or growth inhibiting. More commonly in biological systems both effects or attributes are simultaneously present. We speak of growth inhibiting or facilitating when one clearly predominates. (B) Research does not yet reveal determinants of neutrophils’ role in malignancy–growth facilitation or inhibition or neither. (C) The uniformity of higher NLR being associated with shorter survival across the common cancers, as in Table 1, tends to indicate that growth facilitation predominates. That neutrophils contribute to malignant growth in most cancers seems clear but how exactly they do so is not [4,5,6,7,8,9]. This paper will outline some of the putative pathways.

This paper is a follow up on, and an updating of Draghiciu et al., 2015’s paper and Wang et al., 2020’s paper where they presented data on a range of already-marketed drugs that had an ancillary attribute of lowering NLR [10,11]. TICO includes two of the 21 drugs reviewed by Wang et al. and Draghiciu et al.–a retinoid and tadalafil.

The regimen presented here, TICO, consists of the phosphodiesterase 5 (PDE5) inhibitor tadalafil, used to treat inadequate erections; isotretinoin, the retinoid used for acne treatment; colchicine, a standard gout (podagra) treatment; and the common fish oil supplement omega-3 polyunsaturated fatty acids, hereafter abbreviated simply as omega-3. These, individually or taken all together, would be expected to impose a low side effect burden, pose a very low risk for serious adverse reactions, and be relatively cheap. They each individually have demonstrated the ability to lower the NLR in humans, as in the representative data reviewed below. TICO therefore has potential to convert a high risk high NLR cancer to a lower risk category by virtue of TICO lowering of the NLR.

No other current standard laboratory blood test is as consistently abnormal across the common cancers as is the association between an elevated peripheral blood NLR and shorter overall survival. Table 1 lists representative reviews of this association in 18 common cancers. There are many other similar reviews and meta-studies showing the same shorter cancer survival associated with higher NLR. All these reviews covered hundreds of previous individual clinical studies on this association over the last decade. Several dozen reviews and meta-studies over the last decade have amply documented the general association of higher NLR with shorter survival in a wide range of the common non-hematological cancers [12,13,14,15,16,17,18].

**Table 1:** List of selected, recent, peer reviewed, published meta-studies of research indicating that a higher neutrophil-to-lymphocyte ratio (NLR) is associated with a shorter overall survival in several common cancers.

Cancer Type	Ref.
colorectal	[19]
pancreatic	[20]
NSCLC	[21]
small cell lung cancer	[22]
prostate	[23]
hepatocellular	[24]
cholangiocarcinoma	[25]
breast	[17]
cervix	[26]
epithelial ovarian	[27]
melanoma	[28]
bladder	[29]
sarcoma	[30]
esophagus	[31]
squamous cell	[32]
glioblastoma	[33]
gastric	[34]
renal clear cell	[35]

During the differentiation process, usually in bone marrow, neutrophils’ nuclei evolve from round to banded and then to the common (normal) lobulated morphology [36]. Secretory granules, found mostly in mature lobulated neutrophils, store elastase, myeloperoxidase, cathelicidins, defensins, VEGF, and matrix metalloproteinases, i.e., granulocyte-colony stimulating factor, G-CSF, or granulocyte macrophage-colony stimulating factor, GM-CSF, together referred to as G(M)-CSF, initiate a release process of neutrophils from marrow.

**Figure 1:**
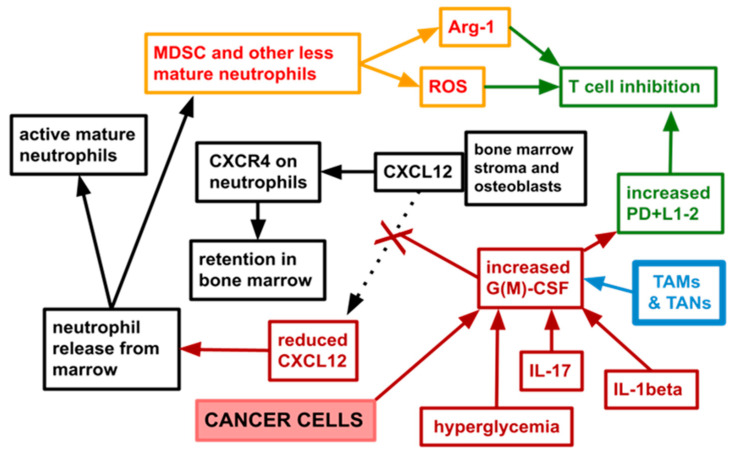
Simplified diagram showing one pathway by which cancer skews the NLR toward neutrophils. References in text. Many steps in the processes of neutrophil retention and release from marrow are not shown here. Many other cytokines not shown here influence and drive both retention and release. G(M)-CSF has many other effects both within marrow and on non-hematopoietic tissues. The depicted action of G(M)-CSF is one among many. The depicted trigger for neutrophil release from marrow, G(M)-CSF, is one trigger among many. Stromal cell-derived factor-1 is now called CXCL12. One of its main receptors is CXCR4. TAMs, tumor associated macrophages, are macrophages or monocyte lineage cells resident within tumors. TANs, tumor associated neutrophils are intratumoral neutrophils. Less mature neutrophils encompasses a subset with T cell suppressing attributes, the MDSC, myeloid derived suppressor cells. MDSC are arginase-1 producing cells that damage nearby T cells.

Only 1 or 2% of the body’s neutrophils reside in circulating blood. A total of 90% reside in bone marrow. The cytokine CXCL12 is expressed in large amounts by marrow stroma. This retains developing neutrophil lineage cells in bone marrow via neutrophils’ surface expression of CXCL12′s receptor CXCR4. G(M)-CSF reduces stromal expression of CXCL12, thereby tending to release the previously held neutrophils [37,38]. See Figure 1 for a simplified diagram of this process.

G(M)-CSF exposure also reduces neutrophil lineage cells’ expression of CXCR4 further contributing to release from marrow. Multiple other cytokines and hormones participate in neutrophils’ exodus or retention from marrow. Multiple counteracting cytokines and hormones act to retain neutrophils in marrow. What happens—release or retention—is the result of the balance between these many forces.

Figure 1 schematically depicts tumor synthesized G(M)-CSF mediating reduction of neutrophil lineage cells’ retention in bone marrow, resulting in an increase in circulating and tumor resident MDSC as well as an absolute neutrophilia. Arg-1 (and other soluble mediators) synthesized by MDSC is locally suppressive of T cells, these processes accounting at least partially for the higher NLR seen across the common cancers.

**Figure 2:**
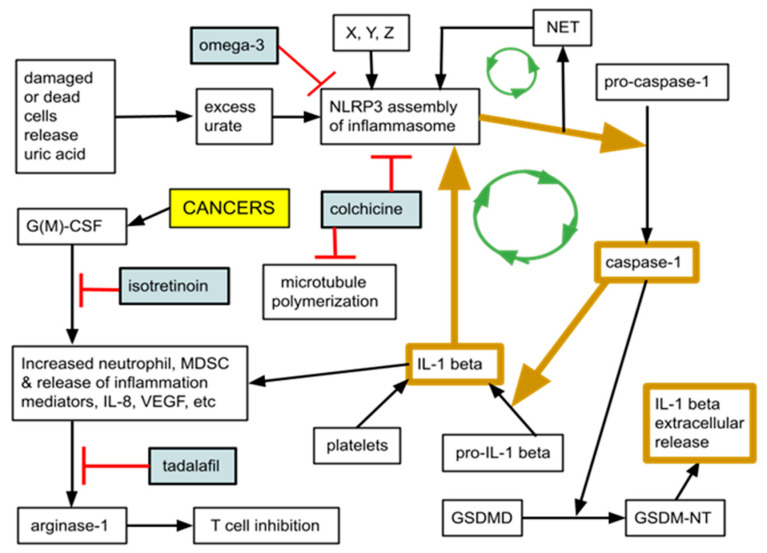
Simplified overview of several of the interacting neutrophil related inflammation systems that in health is crucial in fighting infection but in cancer facilitates malignant growth. Locus of action is also depicted for the TICO drugs tadalafil, isotretinoin, colchicine and omega-3. Intermediate steps are omitted from this schematic, f. ex. isotretinoin probably affects IL-1 beta by suppressing transcription of its mRNA. X, Y, Z refers to a variety of stimuli, including infection, tissue damage, increasing ROS, microbial components, lysosomal rupture, mtDNA, particulate matter, and metabolic dysregulation; GSDMD, gasdermin; MDSC are myeloid derived suppressor cells, a less than fully mature neutrophil subset with normal, mature morphology on H&E. Important but not shown in this schematic, is caspase-1 mediated conversion of pro-IL-18 to active IL-18. Note potential amplification feedback loops between NETs, IL-1beta and the NLRP3 inflammasome. Not shown are multiple inhibiting factors at each step.

There exists a suite of neutrophil related elements that each have a literature database showing their contributions to malignant growth, acting both individually and as an ensemble. This neutrophil centered, interacting, multicomponent inflammation system consists of cancer cell synthesized G(M)-CSF [39,40,41], neutrophil extracellular traps (NETs) [42,43], activation of the NLRP3 inflammasome with consequent increases in MDSC functions [44,45,46], IL-1beta activation [47,48], normal mature neutrophils, and the higher NLR. As an ensemble, these elements function together to fight infection and contribute to wound healing but in cancer become pathologically engaged, facilitating tumor growth and metastasis [49,50]. Core elements of this neutrophil centered system are diagrammed in Figure 2. Table 2 gives quick reference definitions of these elements.

The reviewed data in this paper lead to three conclusions: (1) that MDSC, NLR, and absolute neutrophil count tend to be associated with enhanced tumor growth across the common cancers, and (2) that several well-tolerated, already marketed drugs from general medical practice, the TICO drugs, have evidence that they can lower the NLR and (3) the risk–benefit ratio favors a clinical study of TICO as adjunct to current standard treatments, particularly those using the immune checkpoint inhibitors.

## 2. The NLR

Humans’ normal NLR tends to be about 2, but values between 1 and 3 can be seen in healthy people [51,52,53]. Age and gender have only minor effects on the NLR. NK lymphocyte count tends to vary inversely with the NLR [52].

Absolute numbers of circulating neutrophils are commonly elevated in cancers generally, where higher numbers are associated with shorter survival [13,54,55,56,57]. Several recent reviews together make a strong empirical case for important growth promotion by neutrophils in the common cancers [13,57,58,59,60,61,62,63,64,65]. However, in addition to the relative numbers to lymphocytes, the NLR seems to be determinative even in presence of non-elevated absolute neutrophil numbers. This may reflect an anti-lymphocyte function of a neutrophil subset. Section 4 below outlines how the neutrophil MDSC subset facilitates tumor growth by damaging local T cells.

High NLR also occurs in the common inflammatory conditions—gout, [66]; Crohn’s disease [67]; COVID-19, [68]; SLE, [69]; rheumatoid arthritis, [70]; erosive osteoarthritis [71]; ankylosing spondylitis, [72]; psoriasis and psoriatic arthritis, [73]; ischemia [74]; and others.

Although we have hundreds of primary studies in human cancers showing abnormally elevated NLR, of which Table 1 is representative, we cannot conclude from this that cancer is an inflammatory disease. However, we might be able to conclude that there is some kind of inflammatory process comprising or contributing to the pathophysiology that facilitates malignant growth.

The association of higher NLR with shorter survival holds particularly prominently in cancers treated with immune checkpoint inhibitors, pointing to an association of higher NLR with greater immunosuppression [75,76,77,78,79,80,81,82,83]. As typical examples, Stares et al. reported pembrolizumab (Keytruda™), a pharmaceutical monoclonal antibody to PD-1, used in non-small cell lung cancer gave an overall survival of 8 months if NLR was >5 but 21 months if NLR was <5, and Muhammed et al.’s study in hepatocellular carcinoma treated with various similar checkpoint inhibitors that showed an overall survival of 8 months if NLR was >5 and 18 months if NLR was >5 [81,84]. Multiple other studies of the NLR effect on immune checkpoint inhibitors showed similar results.

Further primary studies on the association of high NLR with lower survival time in these individual cancers have come out since the listed reviews below were published. The listed reviews below summarize hundreds of primary studies documenting that higher NLR correlates with a shorter than average survival in their reviewed cancer. The importance of how uniform this relationship is across multiple cancers is underappreciated in the oncology community. Many concordant references to meta-studies could be listed for each entry but only a single recent review of past work is listed.

## 3. G(M)-CSF

Both G-CSF and GM-CSF are 15 to 20 kDa glycoproteins central to, but not essential for, bone marrow hematopoiesis. G(M)-CSF acts (1) as a direct growth factor acting on cell surface receptors on malignant cells or, (2) as an indirect growth factor by increasing MDSC, or (3) by increasing numbers of normal mature neutrophils that provide trophic and angiogenic factors [3,85,86]. Tumor synthesized G(M)-CSF has been recognized for over a decade now as an element driving immunosuppression in cancer [87].

Common metastasizing cancers tend to be whole body diseases. Abnormal and pathophysiologically relevant bone marrow or spleen hematopoiesis is driven in cancer in part by tumor-synthesized G(M)-CSF [40,41,87,88,89,90,91]. The net tendency of excess G(M)-CSF, whether malignant tissue produced or exogenous as a pharmacological agent, is to hasten transit time through the stages of granulopoiesis, resulting in increased neutrophil release, but also release of less mature neutrophils which are thought to comprise the MDSC subset. Retention in marrow is the result of many factors or retention axises, several of which are disrupted by G(M)-CSF [1,92]. Two of these retention pathways are diagrammed in Figure 1.

However, other cytokines, such as IL-1beta, IL-8, and IL-6, are also involved, and granulopoiesis will proceed even in absence of G-CSF. There may also be immune response reducing effects directly on T cells via a G-CSF receptor pathway on T cells [92].

Multiple stimuli trigger increased G(M)-CSF synthesis by endothelial cells, fibroblasts, macrophages, monocytes, and bone marrow stroma. These stimuli—examples are vascular endothelial growth factor (VEGF), IL-1beta, IL-17, bacterial lipopolysaccharides, TNF-alpha, i.e.,—act via different pathways to result in increased G(M)-CSF. The stimuli for malignant tumor synthesis and secretion have not been established but the correlation between a tumor’s higher production of G(M)-CSF and more aggressive clinical course is well established [40,41,87,88,89,90,91,92,93].

The partially separate matter of monocytic lineage cells’ MDSC will not be discussed here. Malignancy associated increases in circulating MDSC tend to be of granulocyte-MDSC subtype [87,88,89,90,91,92,93].

In several transplantation models G-CSF promoted expansion of MDSCs and enhanced their suppressive function against T cell proliferation [94,95]. Breast cancer cell secreted GM-CSF drove immunosuppression in vitro by increasing MDSC Arg-1 expression thereby inhibiting T cell function, human breast cancer biopsy tissue reflecting this as well [96]. Placental origin G-CSF upregulates systemic MDSC numbers and pharmaceutical G-CSF increases MDSC during transplantation related neutrophil mobilization [97]. Collected data on G-CSF mediated increases in circulating MDSC was recently reviewed [91].

## 4. MDSC

Human MDSC have been variously defined but are commonly thought of as monocytic, M-MDSC CD11b + CD14 + CD15 − CD33 + HLA−DR−, or granulocytic, granulocyte-MDSCs, CD11b + CD14 + CD15 + (or CD66b+) CD33+LOX-1+ [98,99,100,101]. In cancer, 79–80% of MDSC are of the granulocyte-MDSC subset, the remainder are M-MDSC. Elevation of circulating MDSC is a uniform finding in human cancers [102,103,104,105].

Multiple signaling pathways drive MDSC expansion. Examples are signaling chains using VEGF, G(M)-CSF, IL-6, IL-1beta, TLR, high lactate levels, and TNF. Note also the potential for positive feedback loops within the chain of mediators leading to MDSC increases as depicted in Figure 2.

Human MDSCs are without co-expression of the MHC Class-II molecule HLA-DR, expression of which would otherwise be expected in mature myeloid and lymphoid cells. MDSCs are characterized by their predominantly immature state, and their T cell immunosuppressive effects. The exact mechanisms of immune response reduction by MDSC are not completely known but do involve over synthesis of arginase-1 (Arg-1) and inducible nitric oxide synthase that can irreparably damage nearby T cells [100,106,107,108]. Arg-1 mediates the reaction:

arginine + H_2_O → ornithine + urea

There is an intrinsic, direct relationship between an elevated NLR and increased numbers and function of both circulating and tumor-resident MDSC [109,110,111,112,113,114,115,116].

In clinical biopsies, tumor Arg-1 expression is localized to granulocytic myeloid cells [117]. Low numbers of granulocytic-MDSC uniformly predict a longer overall survival during treatment with Is ipilimumab, nivolumab, or pembrolizumab in melanoma [118], in epithelial ovarian cancer [119], and in non-small cell lung cancer [120,121].

Concordant with the notion that NLR and MDSC are positively correlated, lower NLR predicts a better response to, and longer survival during, treatment with immune checkpoint inhibitors in melanoma [122], in non-small cell lung cancer [123,124], in clear cell renal carcinoma [125], head and neck squamous cell carcinoma [126,127], urothelial carcinoma [128,129], cervical cancer [130], and hepatocellular carcinoma [131].

It is the contention of this paper on TICO that, based on data reviewed above, the activated NLRP3 inflammasome, MDSC, G(M)-CSF form a constellation, a triad that is at least partially responsible for the elevated NLR and NETs that we see in cancer. As diagrammed in Figure 1, tumor synthesized G(M)-CSF results in release of greater numbers of MDSC that are both trophic for cancer cells but also T cell suppressing via over synthesis of Arg-1 and other soluble mediators, thereby skewing the NLR to higher ratios. The activated NLRP3 inflammasome is one of the signaling hubs connecting the constellation [46,132,133]. The TICO drugs aim to disrupt this cancer growth enhancing constellation.

## 5. The TICO Drugs

As is the case with the other multi-drug regimens, individual dosing based on tolerance will be required. Clinical study of stepwise addition of the TICO drugs, titrated to tolerance and reduction of NLR in metastatic cancer alongside standard current treatment will tell us if pharmacological lowering of NLR will improve survival or not.

As with all the individual drugs in similar multi-drug regimens, as we outlined in past work, we would not expect any single drug or intervention added to standard treatment to strongly lengthen survival [134,135,136]. As we have outlined previously, absent a “silver bullet” and identification of a single element that drives all the multiple attributes of malignancy, a multidrug approach will be needed to prolong life in the common metastatic cancers [63]. TICO attempts to address one of the several pathological engagements of normally functioning body systems that are pathologically engaged to facilitate growth–in TICO, the bone marrow, and neutrophil related contributions.

The mechanism of action in lowering NLR of TICO regimen drugs have not always been fully delineated. The focus below on TICO drugs emphasizes evidence of their empirical effect on NLR in clinical use more than their mechanism of achieving that reduction.

### 5.1. Tadalafil

Tadalafil is a PDE5 inhibitor, given at 40 mg/day for pulmonary hypertension and 5 mg/day as needed for erectile dysfunction.

In men with severe erectile dysfunction, oral tadalafil 5 mg qd lowered NLR from 1.9 to 1.6, a ~16% reduction [137]. NLR in age-matched controls was 1.3.

After reviewing previous murine studies showing MDSC function was suppressed by tadalafil, Noonan et al. reported a single case study of reduced MDSC function in a 50 y/o patient with IgG kappa multiple myeloma who may have benefited clinically from tadalafil (dose not specified) as part of a multi-drug cocktail [138].

Preoperative oral tadalafil 10 mg/day lowered circulating and intratumoral MDSCs but increased intratumoral CD8+T cells in patients with primary head and neck squamous cell carcinoma [139,140]. Oddly, treatment was given preoperatively only and therefore as could be expected, no clinical benefit was seen.

After studies showing prolonged survival and decreased MDSC function in tadalafil treated melanoma bearing mice, a human study in advanced melanoma shows no/marginal survival prolongation with 10 mg/day tadalafil but did see slight decreases in monocytic MDSC and slight increase in intratumoral CD4 and CD8 T cells [141]. Empirically, tadalafil 20 mg/day lowered blood levels of Arg-1, nitric oxide synthase, MDSC, and Tregs in head and neck squamous cell carcinoma patients [142].

Tadalafil lowered both intratumoral and splenic monocytic and granulocytic MDSC subsets in a murine hepatocellular carcinoma model [143]. Tadalafil decreased tumor Arg-1 and lowered both granulocytic and monocytic MDSC in a murine colon cancer model [144].

Another pharmaceutical PDE-5 inhibitor, vardenafil, also marketed to treat erectile dysfunction and pulmonary arterial hypertension, lowered NLRP3, IL-1beta, active caspase-1, and NLR in experimentally induced murine cholestatic hepatitis [145].

### 5.2. Isotretinoin

Isotretinoin (13-cis-retinoic acid) is a pro-drug for all-trans retinoic acid (ATRA), used in general medical practice to treat pustular acne, primarily in young people. Time-limited isotretinoin treatment can result in long-term remission of acne. Secondary clinical use is as a component of a multi-drug regimen to treat acute promyelocytic leukemia and neuroblastoma.

Oral isotretinoin inhibits sebaceous glands activity. In treating acne, a common isotretinoin dose would be 1 to 2 mg/kg/day for 3–4 months when clearance of inflammatory acne is usual [146,147]. Cheilitis and xerosis are common side effects while photophobia, elevated liver enzymes, decreased appetite, and headaches are seen with less frequency. Minor elevations of cholesterol and particularly triglycerides are common. Serious side effects are rare [148,149].

Isotretinoin is unequivocally teratogenic so strictest birth control measures must be in place. Although safe enough to be used to treat acne in young people, there have been rare reports of fatal reactions to isotretinoin [150].

Over six p450 hepatic enzymes participate in isotretinoin metabolism. As we could expect that results in >10-fold variability in plasma levels even when dosed 160 mg/m^2^ BSA/day, or 5.33 mg/kg for children <12 kg [151]. Isotretinoin is an integral part of current neuroblastoma treatment where target dose is Cmax plasma ≥2 μM. To achieve this requires wide individual oral dose adjustments.

Typical findings of NLR in untreated acne would be 2.1 compared to healthy controls of 1.6 [152]. Isotretinoin at 0.5 to 1 mg/kg/day in severe acne reduced NLR from 1.9 before treatment to 1.8 after three months treatment [153]. Michaëlsson et al. reported NLR decreases in acne cases even at isotretinoin 0.1 mg/kg/day [154].

In young people with severe acne, NLR went from 2.2 to 1.9 after isotretinoin for 90 days [155]. Four similar studies found comparable small decreases in isotretinoin treated acne [156,157,158,159]. However, some found no change in the NLR in isotretinoin treated acne [160].

Retinoids enhance granulocytic differentiation in marrow, reducing release of immature forms, such as granulocyte-MDSC [161]. In vitro cultured human hematopoietic progenitors under G-CSF or GM-CSF exposure generated granulocyte-MDSC that showed reduced Arg-1 content and reduced T cell inhibition ability after addition of ATRA [162].

The T.R.U.E. TEST™ is a proprietary epicutaneous adhesive patch test to clinically determine in vivo degree of ability to develop new delayed cell-mediated hypersensitivity reactions, type IV, immune responses. The panel of patch allergens penetrate skin and are then presented to T helper cells by skin-resident Langerhans cells. After oral isotretinoin for 13 days humans’ ability to become sensitized to T.R.U.E. TEST antigens increased [163].

ATRA reduced immunosuppressive effect of tumor-infiltrating MDSC in vitro and in vivo in a murine cervical cancer model [164]. In 2021 Abrams reviewed evidence of ATRA’s reduction of MDSC [101].

### 5.3. Colchicine

Colchicine is an ancient drug. It is effective, used for centuries and continuing so today (2022) to effectively treat gout (podagra), Familial Mediterranean Fever, Behcet’s disease, aphthous stomatitis, selected cases of pericarditis, and other inflammation related conditions [165,166,167,168].

Colchicine binds to beta-tubulin, hindering its polymerization into microtubules [169]. Binding to beta-tubulin already polymerized in a microtubule promotes microtubule depolymerization, particularly in neutrophils that tend to preferentially concentrate colchicine. The therapeutic oral dose range lies between 0.015 and 0.03 mg/kg corresponding to 1 mg to 2 mg/d per os for a 70 kg human [166]. Fractional nanomolar concentrations of colchicine inhibit neutrophil chemotaxis.

However, colchicine has a narrow therapeutic window. When prescribed daily and chronically for Familial Mediterranean Fever, the most common adverse effects are abdominal pain, diarrhea, nausea, and vomiting. They are usually mild, transient, and reversible on dose lowering [165]. Colchicine overdose results in multiple organ failure and a high mortality. In cases of colchicine poisoning, doses as low as 7 mg have been reported to be fatal. At higher doses colchicine’s action becomes similar to traditional cytotoxic cancer chemotherapy vinca class drugs vinorelbine, vincristine, vinblastine.

Cell surface receptors, selectins, and ion pores’ surface topography are placed and kept in position by intracellular microtubules. Proper positioning of purinergic receptors P2X7 and P2X2 and intracellular assembly of the NLRP3 inflammasome require well-functioning microtubules, processes impeded by colchicine [165,170,171].

Accordingly, colchicine reduces intracellular cleaved (active) caspase-1 protein and of cleaved (active) IL-1beta, without changing mRNA expression of NLRP3 or pro-IL-1beta [172]. This is diagrammed in Figure 2.

In gout (podagra) colchicine administration results in disassembly of the NLRP3 inflammasome and is used in that role in mitigating the cytokine storm of severe COVID-19 [173,174,175].

During an attack free period, people with Familial Mediterranean Fever who were taking colchicine 1 mg/d had NLR of 1.7, while an age matched control group had an NLR of 1.9; however, that difference was considered non-significant at *p* = 0.46 [176].

Colchicine inhibits the synthesis of TNF-alpha, leukotriene B4, prostaglandin E2, TxA2, and impairs adhesion of neutrophils to endothelium by an inhibition of P-selectin expression [177].

In a study of type 2 diabetes, the average NLR was 2.6. This was not meaningfully decreased by a year of colchicine o.5 mg/day [178].

Colchicine, 1.5 mg/d, lowered NLR in Behçet’s disease but took 3 months to reach maximum effect, lowering NLR from 2.5 prior to colchicine, to 2.0 after 30 days, and to 1.7 after 90 days of treatment [179]. In untreated aphthous stomatitis the NLR was 4.5, lowering to 3.9 after 90 days of colchicine 1.5 mg/d [180]. In that study after 90 days of colchicine, the absolute neutrophil count decreased from 4.9 to 3.9, while hemoglobin and hematocrit remained unchanged.

### 5.4. Omega-3 Polyunsaturated Fatty Acids (Omega-3)

Omega-3s are a heterogeneous group of fatty acids with a double bond between the third and fourth carbon atoms from the methyl end (from the ω−1 carbon atom) with fatty acid chains of four to 28 carbon atoms. The aquatic literature quotes mainly two omega-3s–eicosapentaenoic acid, (EPA) C20:5*n*-3 and docosahexaenoic acid (DHA), C22:6*n*-3 [181].

Fish are the primary source of EPA and DHA for humans, but humans can and do synthesize these from plant polyunsaturated fatty acids such as alpha lipoic acid. Marine algae are rich in EPA and DHA.

Recent reviews on how omega-3s might reduce the NLR conclude that none of the data are definitive as to mechanism of action [182,183].

The health-related biologic effects of omega-3s are difficult to interpret due to the many dozen forms that comprise this heterogeneous group of fatty acids. DHA and EPA have been the subject of particular attention.

Some of the variables complicating study of omega-3s: (1) Omega-3s are readily oxidized, changing their biologic effects. (2) Omega-3s from different sources have differing mixes of multiple chain length fatty acids. (3) Research on clinical effects of omega-3s has not always defined the proportions of the various chain lengths used. (4) Even GMP suppliers of omega-3s for human use do not always supply that information. (5) Biologic equivalencies between the various omega-3s have not been determined. (6) Relative ease of in vivo oxidation of the various chain lengths has not been determined. (7) In most jurisdictions around the world, products for human consumption labeled to contain “omega-3” fatty acids are not controlled to the pharmaceutical standards of prescribed medicines so actual omega-3 content is uncertain and variable. However, given these caveats, we do have evidence, outlined below, that omega-3 supplementation in humans can lower NLR.

Excision of dogs’ left atrial appendage raised CRP and the NLR. Post-operative NLR was 50% lower in dogs receiving fish oil supplementation for three weeks prior to surgery with fish oil containing 0.6 g omega-3/kg/day [184]. A corresponding intake in humans might be hard to achieve or difficult to tolerate.

Preimmunized mice with antigen induced peritonitis who were fed dietary fish oil prior to peritoneal antigen challenge had increased recruitment of the most mature subtype of NK cells compared to control mice [185]. Neutrophil counts in their peritoneal fluid were half those in controls. The fish oil used was 2.8% menhaden fish oil (Omega Protein™, Reedville, VA, USA) containing 15% of EPA and 11% of DHA, resulting in 4.0 g EPA and 2.9 g DHA per kg diet.

Medical students given 2.5 g/d, 2085 mg EPA and 348 mg DHA (OmegaBrite^TM^, Waltham, MA, USA) or placebo capsules resulted in plasma levels 6-fold higher of EPA and 50% higher DHA compared to levels prior to supplementation [186].

Omega-3s interfere with the NLRP3 inflammasome assembly, thereby reducing activated (matured) IL-1beta [187,188,189,190].

In healthy adults without overt evidence of inflammation, the omega-3 level was inversely related to the NLR [173]. In a study of 28,000 individuals, McBurney et al. found an NLR of 2.1 in those when omega-3s comprised less than 6.6% of cellular fatty acids and an NLR of 1.9 in those with omega-3 comprising greater than 6.6% [190].

Patients with longstanding painful knee joint osteoarthritis had a NLR of 1.8 that decreased to 1.5 after six months of krill oil supplementation (4 g krill oil/d, 0.60 g EPA/d, 0.28 g DHA/d, 0.45 g astaxanthin/d) [191].

In a study of acute-on-chronic liver failure patients, Kulkami et al. found a NLR of 6.9 on hospital entry, decreasing to 3.6 after a week supplementation with 100 mL/day (112 Kcal) of 10% Omegaven™ (Lake Zurich, IL, USA), an omega-3 rich oral emulsion, while remaining unchanged (at 6.9) in those receiving an equicaloric lipid emulsion without omega-3 [192].

## 6. Discussion

A pilot study of the TICO regimen would be a step in determining if high NLR indeed facilitates malignant growth or is a non-contributing consequence of malignant growth. The TICO regimen was developed by combining older, non-oncology medicines that have demonstrated clinical ability to lower the NLR in humans.

Multiple other FDA approved drugs also have data showing NLR reduction. For example, and of great potential importance, Baker et al. in 2008 demonstrated significant reduction in NLR by sulfadoxine-pyrimethamine (Fansidar™) [193]. Vitamin D has a dozen papers showing reduction in NLR with vitamin D supplementation [194,195,196]. Several other FDA approved drugs also have a database showing NLR lowering in humans. The TICO drugs were selected based on a risk–benefit assessment considering expense, adverse effect profiles, and potential pharmacologic interactions with each other and with commonly used standard medicines in oncology.

None of the TICO drugs seem individually to provide robust NLR lowering. The only easily foreseeable way to determine if pharmacologically changing a high NLR to a low NLR will lengthen survival in the common metastatic cancers is to try it.

Many open questions about adjunctive use of a multi-drug regimen such as TICO in treating cancer require answering. Are the drugs of TICO additive? Are any of the drugs detracting from the effects of any of the other drugs? Will medically lowering NLR have clinical benefit similar to the patient subset having untreated low NLR?

A much-increased NLR is seen following any process that damages brain tissue by any means—stroke, ischemia, metastasis, aneurysm, multiple sclerosis, encephalitis, or surgical or traumatic brain tissue severing. The NLR usually increases after severe closed head trauma even in absence of overt MRI evidence of gross tissue damage or bleed [197,198,199,200,201,202,203,204]. These are elements contributing to systemic immunosuppression that is seen following severe closed traumatic brain injury [205]. We would expect this to happen, given G(M)-CSF’s role as a neurotrophic factor, functioning within the brain entirely independently from their effects on marrow and hematopoiesis, combined with the increases in MDSC after large pulses of G(M)-CSF.

We discussed and reviewed past research data on the elements of neutrophil related growth enhancement during the course of cancer: the (1) increases in MDSC, (2) neutrophil NLRP3 inflammasome activation, (3) increased NETosis, (4) increased absolute neutrophil count, and (5) higher NLR. These together form an integrated, related, interacting system that each individually or as an ensemble contribute to malignant growth across the common cancers.

## 7. Conclusions

Higher NLR is a well-established prognostic indicator of shorter overall survival across the common metastatic cancers. MDSC, NLRP3 inflammasome, NETosis, and absolute neutrophil count are related to the NLR. They, individually and as an ensemble, contribute to cancer growth and metastasis. The regimen presented in this paper, TICO, was designed to decrease these elements of malignant growth. The drugs of TICO are old, cheap, available worldwide, and when used in their non-oncology indications, carry an acceptable risk of side effect burden. They all have evidence of lowering the NLR or the growth contributing elements related to the NLR when clinically used in general medicine as reviewed in this paper.

## Figures and Tables

**Table 2:** Neutrophil related interacting elements that are currently recognized as contributing to malignant growth. For references see the corresponding main text section.

Acronym	Description
ANC	absolute neutrophil count as determined on the standard complete blood count.
MDSC	myeloid derived suppressor cells–are divided into those with monocytic features on H&E staining, M-MDSC, CD14+ HLA-DRlow/CD15 cells, and those with neutrophil features on H&E staining, granulocyte-MDSCs, CD11b+CD14+ CD15+ (or CD66b+) CD33+LOX-1.
NET.	neutrophil extracellular trap–neutrophil extracellular traps are web-like structures, usually, but not always, extracellular and intravascular, containing decondensed DNA from neutrophils, histones, cathepsins, neutrophil elastase, myeloperoxidase, and multiple other neutrophil granule proteins.
NLR	neutrophil-to-lymphocyte ratio as determined on the standard complete blood count.
NLRP3	nucleotide-binding oligomerization domain, leucine rich repeat and pyrin domain containing 3, a cytosolic 118 kDa protein forming a central component of a macromolecular assembly, the NLRP3 inflammasome. NLRP3 is a 118 kDa cytosolic protein normally found in neutrophils, monocyte lineage cells, neurons and other cells. NLRP3 oligamerizes and associates with a set of other proteins to form the NLRP3 inflammasome that in turn mediates conversion of pro-caspase-1 to catalytically active caspase-1 that in turn catalyzes several inflammatory cytokines’ precursor forms to their active signalling forms. In the current literature “NLRP3” is sometimes used to refer to the entire oligomeric inflammasome complex and sometimes used to refer to the 118 kDa core protein alone. NLRP3 inflammasome is activated by many diverse triggers. Examples: uric acid crystals, silica particles, microscopic asbestos fibers, extracellular ATP, assorted toxins, common motifs of viruses, bacteria, fungi, and protists.

## Data Availability

All new data has been presented in this paper. There is no further data but the author welcomes questions and discussion.

## Abbreviations

All-trans retinoic acid (ATRA); arginase-1 (Arg-1); docosahexaenoic acid (DHA); eicosapentaenoic acid (EPA); G-CSF or GM-CSF, together referred to as (G(M)-CSF); myeloid-derived suppressor cells (MDSC); neutrophil extracellular traps (NETs; neutrophil-to-lymphocyte ratio (NLR); phosphodiesterase 5 (PDE5); the NLR lowering regimen, tadalafil, isotretinoin, colchicine, omega-3 fish oil (TICO); vascular endothelial growth factor (VEGF).
